# Histone deacetylase 2 and 3 of *Sarcoptes scabiei*: characterization of a potential drug target

**DOI:** 10.1128/spectrum.00737-24

**Published:** 2024-10-22

**Authors:** Ran He, Luyang Xu, Maochuan Guo, Kai Cheng, Ziyi Song, Yue Xie, Hui Wang, Xuan Zhou, Xiaobin Gu, Jing Xu, Huidan Deng, Guangyou Yang

**Affiliations:** 1 Department of Parasitology, College of Veterinary Medicine, Sichuan Agricultural University, Chengdu, China; Hubei University of Medicine, Shiyan, Hubei, China

**Keywords:** scabies, HDAC, deacetylation assays, drug target

## Abstract

Scabies is a contagious zoonotic parasitic disease that causes a substantial risk to both human and animal health and results in significant financial losses. No vaccine is available for scabies, and drug resistance to the conventional treatment for the disease has increased. Histone deacetylase (HDAC) modifies proteins by removing acetyl moieties from histones, regulates transcription, and is crucial for the immune system and apoptotic processes. This study aimed to clone, express, and determine the immunoreactivity of HDAC-2 and HDAC-3 of scabies mites to investigate their potential as scabies drug targets. The effects of inhibitors on recombinant *Sarcoptes scabiei* HDAC-2 (rSsHDAC-2) and rSsHDAC-3, as well as on the survival rate and ultrastructure of scabies mites *in vitro*, were also verified. The findings showed that the inhibitors reduced the acetylase activity of rSsHDAC-2 and rSsHDAC-3. Additionally, these inhibitors could significantly reduce the survival rate of scabies mites, making structural alterations in the mites such as mitochondrial pyknosis and cytoplasmic vacuoles and reaching a fatality rate of 76.7% after 24 h of action. In conclusion, HDAC-2 and HDAC-3 were critical to the survival of scabies mites and might be targeted by medications. Furthermore, the effect of inhibitors on the survival rate and structure of isolated scabies mites provides a new direction for developing therapeutic drugs for scabies.

**IMPORTANCE:**

In this study, we successfully cloned and expressed recombinant SsHDAC-2 and SsHDAC-3 in a prokaryotic system and confirmed their acetylation-deacetylase activities. These results provide a solid experimental foundation for subsequent research on SsHDAC-2 and SsHDAC-3. Furthermore, we report for the first time the use of SsHDAC-2 and SsHDAC-3 as drug targets. We demonstrated that the inhibition of these HDACs by pharmacological agents can lead to structural damage in the parasite, thereby impacting the survival activity of the scabies mite. This finding opens up a novel therapeutic avenue for the treatment of scabies.

## INTRODUCTION

Scabies is one of the most common skin diseases in the world, which is caused by scabies mite infection. It has a diverse host range and affects more than 150 species of mammals worldwide, including humans, domestic animals, and wild animals (1). Around 300 million people are infected by scabies mites annually worldwide, with incidence rates varying from 0.2% to 71.4% in different geographical areas. The World Health Organization has classified it as a neglected tropical disease (2). The clinical signs of scabies mite infection include itching, weight loss, hyperkeratosis, and inflammatory skin lesions in the host. Scabies has an adverse effect on the welfare and productivity of animals, increasing the mortality and causing large financial losses (3). No vaccination is available for scabies, and the infection is mostly treated with medications. Drug resistance to the most commonly used medications, such as ivermectin, has increased (4). Therefore, discovering new pharmacological targets has become a priority.

Histone deacetylase are enzymes that remove acetyl groups from lysine residues through epigenetic posttranslational modification of histones (5). HDAC can condense chromatin and impede gene transcription (6). It can also modulate chromatin function via a complex regulatory network of histone and nonhistone substrates (7). HDAC is expressed at various developmental stages of many parasites and plays an important role in controlling stage-specific gene expression during parasite differentiation (8). Therefore, HDAC inhibitors are currently being used to investigate their inhibitory effect on parasite survival and development. Broad-spectrum HDAC inhibitors are currently being used to treat parasites such as protozoa, flukes, and tapeworms (9, 10). These inhibitors can disrupt several cellular processes and increase toxicity. However, sub-type selective inhibitors are implicated in specific disease mechanisms and have lower degrees of cytotoxicity (11). HDAC-3 belongs to class I HDAC, which is widely expressed in organisms, participates in many physiological processes, and has been used as a drug target for many diseases. SsHDAC-2 has high homology with HDAC-11 of other species through sequence alignment. HDAC-11 is mainly located in the nucleus and plays an important role in various tumor diseases, biological barriers, lipid metabolism, genome stability, and cell cycle progression (12). Consequently, it is crucial to investigate the potential of SsHDAC-2 and SsHDAC-3 as acaricidal antigens for treating scabies.

## MATERIALS AND METHODS

### Mites serum animals

*S. scabiei* mites were provided by the Department of Parasitology at Sichuan Agricultural University. Different life stage mites (adult, nymphs, and larvae) were separated and collected after morphological identification. Six healthy New Zealand rabbits (eight weeks old, female, 3–3.25 kg) and four Sprague-Dawley rats (six weeks old, female, 150–200 g specific pathogen-free) were purchase from Dashuo Laboratory Animal company (Chengdu) and SPF Biothchnology company (Beijing) respectively.

### SsHDAC-2 and SsHDAC-3 mRNA levels in *S. scabiei* mites

Real-time quantitative polymerase chain reaction (RT-qPCR) analysis was used to verify the mRNA expression levels of SsHDAC-2 and SsHDAC-3 at different developmental stages of scabies mites, and β-actin was used as the internal reference gene. Utilizing an RNA extraction kit from Tiangen (Beijing, China), total RNA from mites (adults, nymphs, and larvae) was extracted, and the RNA quality and quantity were assessed using the 1.5% agarose gel electrophoresisthe and the NanoDrop 2000 spectrophotometer. An amount of 1 µg of total RNA was reverse transcribed to cDNA using the RevertAi First Strand cDNA Synthesis Kit (Thermo Fisher Scientific, Waltham, MA, USA) according to the manufacturer’s instructions. The nucleic acid sequences of SsHDAC found in NCBI (SsHDAC-2: KAF7495531.1, SsHDAC-3: KAF7495820.1) were used as templates to create qPCR primers (Table S1), which were then synthesized for qPCR using the LightCycler480 System (Roche Diagnostics). The reaction mix (20 µL) includes SYBR Permix Ex TaqII (FOREGENE, Chengdu, China) 10 µL, upstream primer 0.8 µL, downstream primer 0.8 µL, cDNA 2 µL, and ddH2O 6.4 µL. The cycling conditions were as follows: an initial denaturation at 95°C for 30 s, followed by 40 cycles at 95°C for 5 s and 60°C for 30 s, and then melting curve analysis at 95°C for 5 s, 60°C for 60 s, and 95°C for 1 s. qPCR was conducted in triplicate, and the relative gene expression levels were calculated using 2^−ΔΔCt^ method (13).

### Bioinformatics analysis

Sequencing results were compared to the SsHDAC-2 and SsHDAC-3 sequences retrieved from the NCBI database using DNAMAN 3.0 to verify their complement. Submit the amino acid sequence of SsHDAC-2 and SsHDAC-3 to the ExPASY online database to predict their molecular weight and electric point. SignalP 5.0 (http://www.cbs.dtu.dk/Services/SignalP/) was used to forecast its signal peptide, and TMHMM-2.0 (http://www.cbs.dtu.dk/services/TMHMM-2.0) was used to predict its transmembrane region. SOPMA (https://npsa-prabi.ibcp.fr/cgi-bin/npsa_automat.pl?page=npsa_sopma.html). Furthermore, the two proteins and their homologous genes were multiple sequence aligned using EMBL-EBI, and a phylogenetic tree was created using the Neighbor-Joining method using MEGA software (version 7.0.26).

### Preparation of SsHDAC-2 and SsHDAC-3

Total RNA from *S. scabiei* (larvae, nymphs, and adults) mite was isolated and reverse transcribed previously, the cDNA was used as PCR template. Primers were designed using the sequence from NCBI (Table S2). PCR products were separated and cloned into the PMD19-T vector (TaKaRa, Beijing, China) and subcloned into the pET32a (+) vector (Novagen, USA) using restriction sites (underlined in the above primers), and the recombinant plasmids were sequenced (Shanghai, China). The identified recombinant plasmid was transformed into *E. coli* BL21 (DE3) cells (Invitrogen, USA), and 1 mM isopropyl β-d-1-thiogalactopyranoside was used to induce the expression of cultured cells. The recombinant protein was purified by chromatography with a Ni-NTA His-tag affinity kit (Bio-Rad, USA) according to the manufacturer’s instructions. Recombinant protein was analyzed for purity by SDS-PAGE using a 12% gel and subsequently with a bicinchoninic acid protein assay kit (Pierce, USA) to estimate protein concentration (14).

### Western blotting

To prepare rat anti-rSsHDAC-2/HDAC-3 IgG, four Sprague–Dawley rats were divided into two groups of two rats each randomly, the purified rSsHDAC-2 and rSsHDAC-3 were used as antigens for immunization, respectively. The recombined protein was emulsified with Saponin adjuvant (Sigma, USA), and 300 µg of recombinant protein was injected subcutaneously. Subsequent subcutaneous injections of 300 µg of recombinant protein were performed on days 7, 14, and 21. Anti-sera from rats was collected 1 week after the final immunization, and the titer of antibody was investigated by ELISA (15). The Protein G-Sepharose column (Bio-Rad, USA) was used to isolated IgG from the collected anti-serum according to the manufacturer’s instructions.

rSsHDAC-2 and rSsHDAC-3 protein samples were boiled for 12 min in electrophoresis sample buffer and then separated by the same 12% SDS-PAGE gel. An electrophoretic transfer cell (Bio-Rad, USA) was used to transfer the protein onto a PVDF membrane, and then the membrane was cut into individual lanes for further analysis. The membrane was incubated with blocking buffer (5% skim milk) for 2 h, followed by serum samples (*S. scabiei* negative and positive rabbit serum, rat negative serum, and rat anti-SsHDAC-2/SsHADC-3 IgG, respectively; diluted 1:100 in PBS) at 4°C overnight. After washing three times with TBS-Tween 20, the membrane was incubated with horseradish peroxidase (HRP)-conjugated goat anti-rabbit IgG antibody (1:3,000; Boster, China) for 2 h. The Enhanced HRP-DAB Chromogenic Substrate Kit (Tiangen, China) was used to visualize the protein signal according to the manufacturer’s instructions (16).

### Immunohistochemical analyses

*S. scabiei* mites were collected and fixed in 1% molten agarose and set in paraffin wax after solidification of the molten agarose. Then mite sample was fixed overnight with a 4% paraformaldehyde fixative (Biosharp, Guangzhou, China). The paraffin-embedded skin samples were sectioned to 5 µm and dried in a 37°C oven for 24 h. Purified rat anti-SsHDAC-2/SsHADC-3 IgG serum and rat negative serum were used as primary antibody (diluted into 1:400); FITC Goat Anti-Rat IgG (H + L) (1:400) (ABclonal, Wuhan, China) were used as Secondary antibody to conduct immunofluorescence localization experiments in *S. scabiei* mites, respectively, to determine the distribution of SsHDAC-2/SsHADC-3 in *S. scabiei* mites (17).

### Deacetylation assays *in vitro*

Refolded rSsHDAC-2 and rSsHDAC-3 protein concentrations were measured and adjusted to 1 mg/mL. The deacetylation assays *in vitro* of recombinant protein were assessed according to Huang (18), and all solutions were prepared with HEPES buffer (25 mM HEPES, 137 mM NaCl, 1 mM MgCl_2_, and 2.7 mM KCl, pH 8.0). The 96-well black plate was used to quantify the deacetylation assays. The study had three groups, each group had three replicate wells. Each group added 10 µL of HEPES buffer, 15 µL of recombinant protein, 25 µL of Boc-Lys(ε-Ac)-AMC (GlpBio, USA) substrate, and 15 µL PBS was added instead recombinant protein in the negative control group. The total system is 50 µL and was cultivated for 2 h at 37°C. Add 50 µL of stop solution [10 mg/mL trypsin and 10 µM TSA (GlpBio, USA)] after the culture, incubate at room temperature for 1 h, and measure the fluorescence value on the multifunctional microplate reader SpectraMax M2 at excitation light 355 nm and emission light 460 nm.

### Deacetylation inhibition assay

Elevenostat and RGFP966 (GlpBio, USA) were diluted to 10 µM, 1 µM, 100 nM, and 10 nM with deacetylation assays buffer for evaluating the inhibitory effects of Elevenostat and RGFP966 on rSsHDAC-2 and rSsHDAC-3, respectively. To make a substrate buffer with a concentration of 100 µM, mix 990 µL of deacetylation assays buffer with 10 µL of the substrate Boc-Lys(-Ac)-AMC. The experiment was carried out in a black 96-well plate with 11 groups, each of which had three replicate wells. In the experimental group, add 1 µL of the matching inhibitor with a different concentration, 15 µL of recombinant protein, 25 µL of substrate, and 9 µL of deacetylation assays measurement buffer. Fifteen microliters of PBS was used in place of the recombinant protein in the negative control group, and no matching inhibitor was added to the blank control group. Incubate for 2 h at 37°C. Add 50 µL of stop solution [10 mg/mL trypsin and 10 µM TSA (GlpBio, USA)] after the culture, incubate at room temperature for 1 h, and measure the fluorescence value on the multifunctional microplate reader SpectraMax M2 at excitation light 355 nm and emission light 460 nm. Three independent assays, each in duplicate, were carried out for the SsHDAC-2 and SsHDAC-3 deacetylase assay with results expressed as percent inhibition of SsHDAC-2 and SsHDAC-3 enzymatic activity (±SD) compared to DMSO vehicle controls (19).

### Acaricidal activity of inhibitors *in vitro*

*S. scabiei* mites were isolated from infected rabbits and used for testing. Crust form the infected skin were collected in the glass plates and incubated at 36°C for 30 min. Live mites that come out from the crust were collected after morphological identification. Experimental procedures were performed as previously described (20, 21).

The RGPF966 and Elevenostat were diluted in 0.25 mg/mL, 0.5 mg/mL, and 1 mg/mL with 10% DMSO, respectively. Two-hundred microliters aliquot of each concentration of the inhibitor was added to six-well culture plates containing filter paper chips to absorb the liquid. Ten *S. scabiei* mites were placed on the filter paper in each well, and the plates were incubated at artificial climate incubator (25°C with a relative humidity of 80%). Check the activity of scabies mites every 2 h until 24 h, and the viability of the mites was checked regularly by needle stimulation, mites that displayed no reaction were recorded as dead. Three petri dishes containing 10% DMSO were used as the negative control.

### Acaricidal activity on different developmental stages mites

The experiment procedure adheres to the guidelines from the previous section. Except that the live mites were collected after morphological identification in different stages (larvae, nymphs, and adults), and the working concentration of RGPF966 and Elevenostat was decided according to the results of the previous section.

### Transmission electron microscopy observation

Transmission electron microscopy (TEM) assay was performed to identify the effects of RGPF966 and Elevenostat on the ultrastructure of mites. Mites were collected in polystyrene plates; the RGPF966 and Elevenostat was diluted with 10% DMSO and added to the polystyrene plate for pretreatment, respectively. Moreover, 10% DMSO was used as negative control. Then, the plates were incubated in an artificial climate incubator (25°C with a relative humidity of 80%) for 12 h. After washed three times with PBS, the mites were fixed by 3% glutaraldehyde and followed by 1% osmic aced and then dehydrated with a series concentration of acetone. Ultrathin longitudinal sections (60–90 nm) were cut, stained with uranyl acetate for 10 min, and lead citrate for 1 min in room temperature. The ultrathin sections were observed by a TEM (JEM-1400FLASH, Japan) (22).

### Data analysis

The data were analyzed using GraphPad Prism 9 software, and survival curves were calculated using the Kaplan-Meier method. Measurement data are expressed as mean ± standard deviation (*X* ± SD, *n* = 3). Comparisons between multiple groups were performed by one-way analysis of variance in IBM SPSS Statistics 20.0. *P* < 0.05 indicated that the difference was statistically significant.

## RESULTS

### qPCR analysis of stage-specific SsHDAC-2 and SsHDAC-3 relative transcription

**Fig 1:**
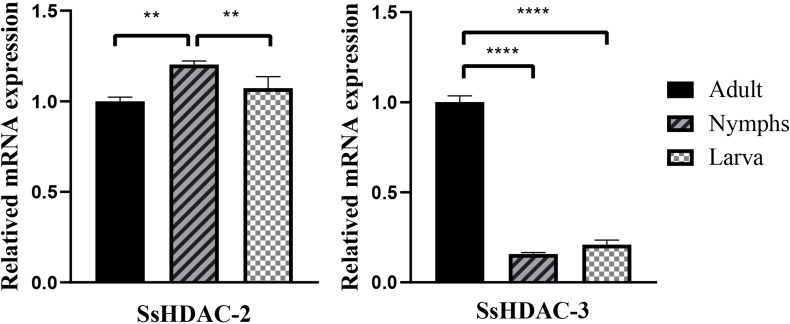
Transcriptional levels of *Ss*HDAC-2和*Ss*HDAC-3 in *Sarcoptes scabiei* different developmental stages. “*” is the expression level of treatment group compared with Control group (*P* < 0.05*，*P* < 0.01**，*P* < 0.001***，*P* < 0.0001****).

Comprehensive analysis using Ssβ-actin as the reference gene showed that the relative transcription levels of SsHDAC-2 and SsHDAC-3 were different at each stage. The relative transcription level of SsHDAC-2 was higher in nymphs than that in larvae and adult mites (*P* ＜ 0.01), and the relative transcription level of SsHDAC-3 was higher in adult than that in larvae and nymph mites (*P* ＜ 0.0001) (Fig. 1).

### Bioinformatics analysis

The open reading frame showed that the SsHDAC-2 and SsHDAC-3 gene sequences contain 915 and 1,236 bp and encoded 304 and 411 amino acids, respectively. The relative molecular weight of SsHDAC-2 and SsHDAC-3 was 51.6 and 63.3 kDa, and the pI was 5.14 and 4.97, respectively. The SsHDAC-2 and SsHDAC-3 contained no signal peptides or transmembrane regions. The SsHDAC-2 shared 46.5%, 41.2%, and 40.2% similarity with the HDAC-11 of *Dermatophagoides pteronyssinus*, *Dermatophagoides farina,* and *Tyrophagus putrescentiae*, respectively (Fig. S1). The SsHDAC-3 shared 76.2%, 75.9%, and 65.6% similarity with the HDAC-3 of *Dermatophagoides pteronyssinus*, *Dermatophagoides farina,* and *Tyrophagus putrescentiae*, respectively (Fig. S2). Phylogenetic tree showed that SsHDAC-2 and SsHDAC-3 shared the same branch with *Dermatophagoides pteronyssinus*, *Dermatophagoides farina,* and *Tyrophagus putrescentiae* (Fig. S3 and S4).

### Expression and identification of SsHDAC-2 and SsHDAC-3

**Fig 2:**
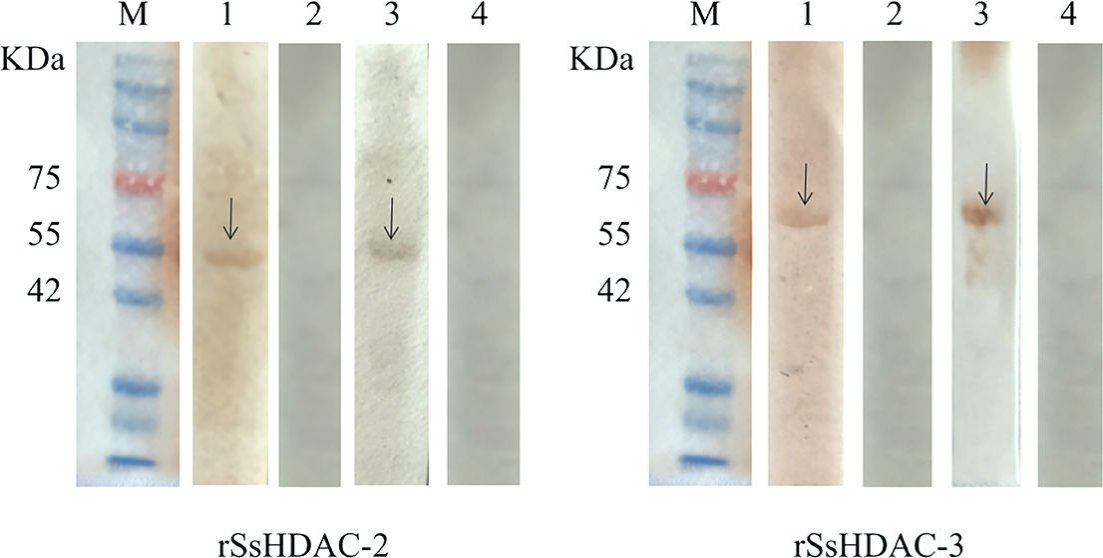
Identification of rSsHDAC-2 and rSsHDAC-3. M: protein marker (common marker lane for rSsHDAC-2 and rSsHDAC-3); lane 1: positive serum from rabbits infected with *Sarcoptes scabiei* react with rSsHDAC-2 and rSsHDAC-3; lane 2: negative serum from uninfected *Sarcoptes scabiei* rabbits react with rSsHDAC-2 and rSsHDAC-3; lane 3: rat polyclonal antibody react with rSsHDAC-2 and rSsHDAC-3; lane 4: rat negative serum react with rSsHDAC-2 and rSsHDAC-3.

The SsHDAC-2 (GenBank: PP510698) and SsHDAC-3 (GenBank: PP516249) genes were successfully cloned from the cDNA of the mixed sample (larvae, nymph, and adult mites) with specific primers. The size was around 900 and 1,000 bp, respectively. Through cloning and sequencing analysis, the amplified gene fragment showed nearly 100% sequence similarity to the sequences present in the transcriptome data of *S. scabiei.* The SsHDAC-3 presented as soluble proteins, while the SsHDAC-2 presented in inclusion bodies. SDS-PAGE analysis revealed that SsHDAC-2 and SsHDAC-3 were purified as clear and single bands at location 51 and 63 kDa (including 18 kDa His-tag protein) (Fig. S5), respectively. The SsHDAC-2 and SsHDAC-3 were specifically recognized by *S. scabiei* positive rabbit serum and anti-SsHDAC2/SsHDAC-3 IgG, but no band was observed after incubation with *S. scabiei* naive rabbit serum (Fig. 2).

### Immunolocalization of SsHDAC-2 and SsHDAC-3

**Fig 3:**
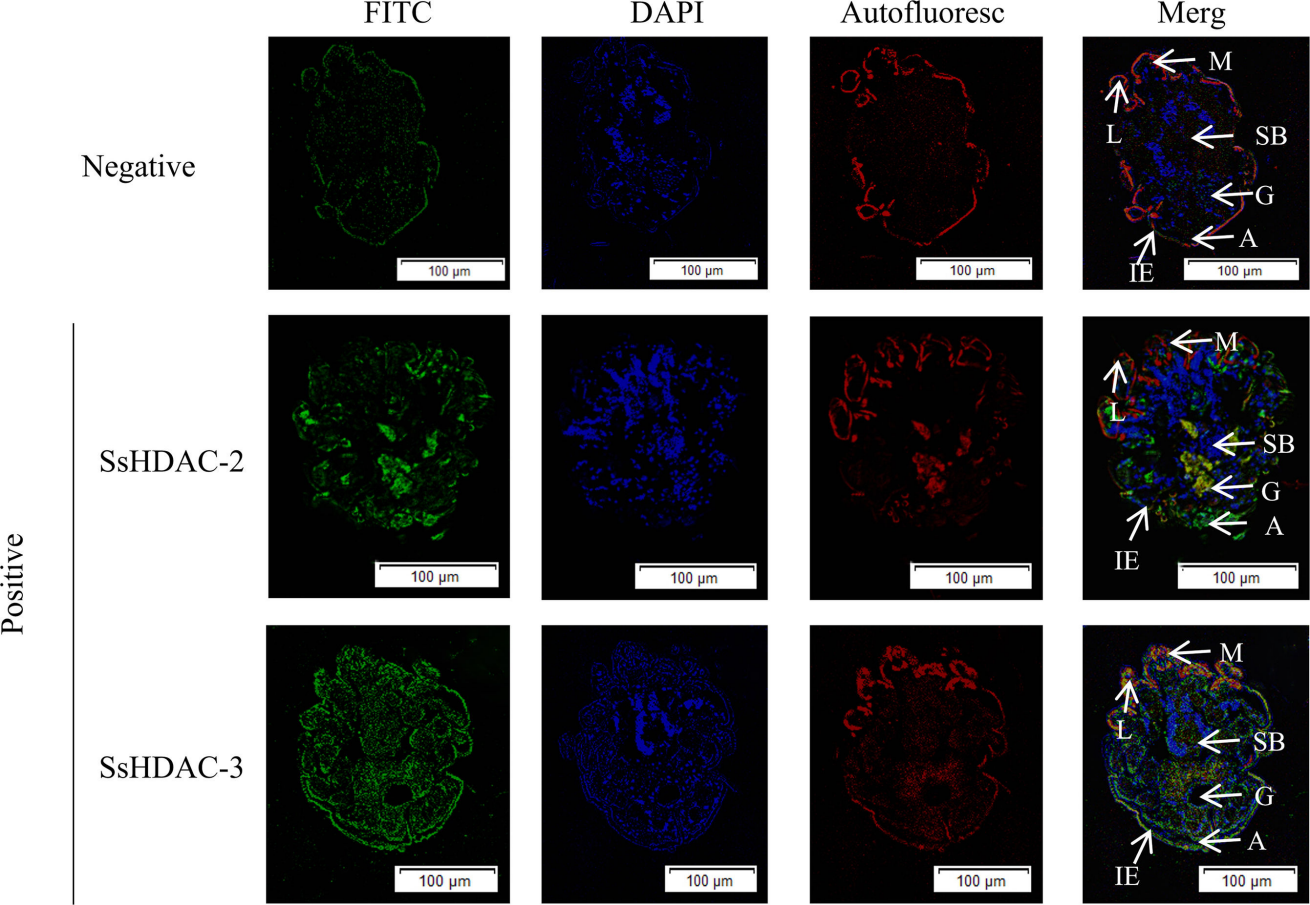
Immunofluorescence localization of *Ss*HDAC-2 and *Ss*HDAC-3 in *S. scabiei*. Positive: Incubated with rat anti-rSsHDAC-2 and anti-rSsHDAC-3 antibody as the primary antibody, Negative: Incubate with rat negative serum as the primary antibody. *Notes*: M, mouthpart; L, leg; G, gut; SB, stomach blocks; IE, epidermal integument; A, anus.

Green fluorescence signals were observed in the gut, stomach, and anus of the *Sarcoptes scabiei* mites using purified rat anti-SsHDAC-2 IgG serum. However, the green fluorescence signals were distributed in and on the body of *S. scabiei* mites using purified rat anti-SsHDAC-3 IgG serum (Fig. 3).

### Deacetylation assays of rSsHDAC-2 and rSsHDAC-3 *in vitro*

**Fig 4:**
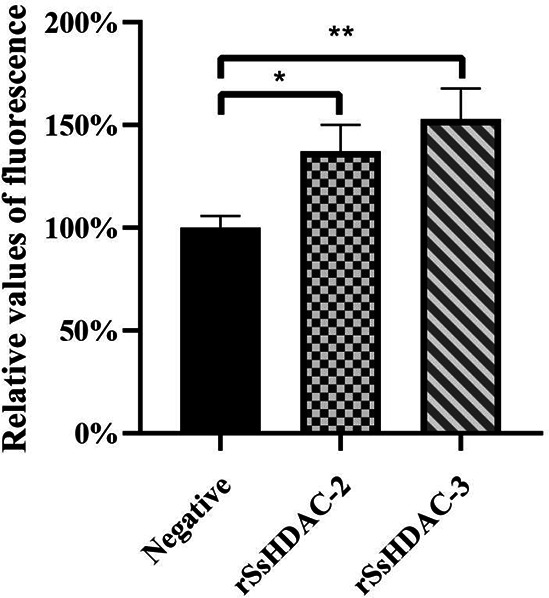
Relative fluorescence intensity values for rSsHDAC-2 and rSsHDAC-3 deacetylation assays *in vitro*. “*” is the relative fluorescence intensity of the experimental group compared to the negative control group (*P* < 0.05*, *P* < 0.01**).

Boc-Lys(ε-Ac)-AMC serves as a substrate for the assessment of HDAC deacetylation assays in *vitro*. AMC contains a fluorophore that is quenched when it is covalently coupled. Upon deacetylation of the substrate, trypsin can cleave the complex, releasing the AMC group. This liberated AMC group can then be excited and will fluoresce. The fluorescence intensity increased (0.01 ＜ *P* ＜ 0.05) in the rSsHDAC-2 group compared with the negative control group, whereas the intensity in the rSsHDAC-3 group increased significantly (0.001 ＜ *P* ＜ 0.01) (Fig. 4).

### Deacetylation inhibition assay

Elevenostat and RGFP966 had an increased inhibitory impact on rSsHDAC-2 and rSsHDAC-3 at increasing concentrations. Elevenostat inhibited rSsHDAC-2 by 81.2%, 47.6%, 32.8%, and 12.3% at concentrations of 10 µM, 1 µM, 100 nM, and 10 nM, respectively. RGFP966 inhibited rSsHDAC-3 by 68.9%, 18.3%, 11.9%, and 5.9% at similar concentrations, respectively (Fig. S6).

### Acaricidal activity of inhibitors

**Fig 5:**
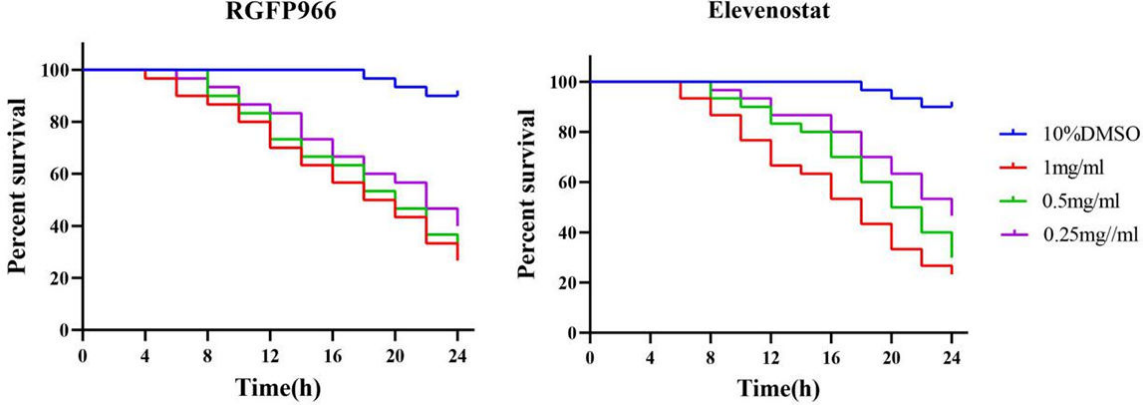
Survival curve of isolated scabies mites under different concentration of RGFP966 and Elevenostat.

The survival rate of isolated mites declined with the increase in the concentration of RGFP966 although no significant difference was observed in the mite inventory rate after the effect of different concentrations of RGFP966. Similarly, as the elevenostat concentration increased, the survival rate of isolated mites decreased. However, the mortality rate of scabies mites after 24 h of action was 53.3% at a concentration of 0.25 mg/mL of elevenostat, and 76.7% at a concentration of 1 mg/mL, indicating a significant difference between the two (*P* ＜ 0.05). Figure 5 displays the percentages of survival of isolated mites given varying inhibitor treatments. The negative control group had no significant influence on the survival of scabies mites, and the mortality rate of these mites after 24 h of treatment was only 10%.

### Acaricidal activity on different developmental stages of mites

**Fig 6:**
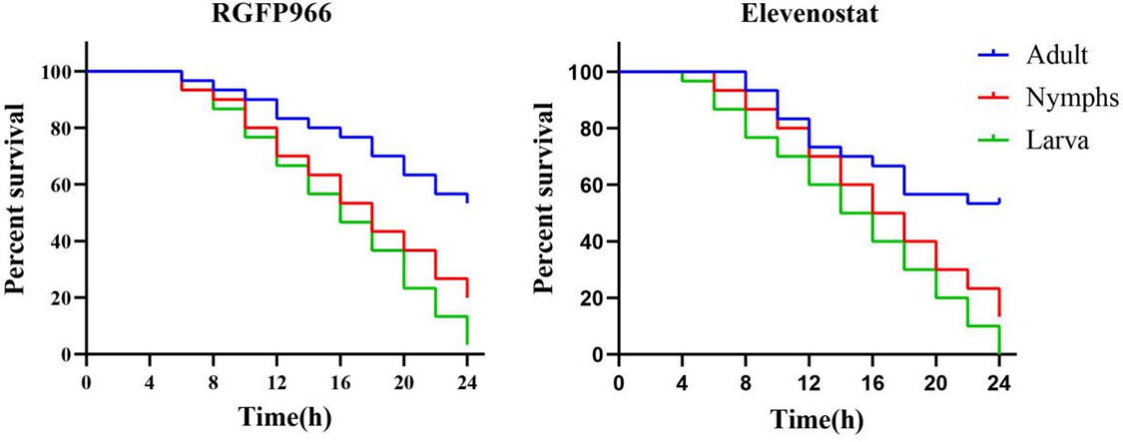
Survival curves of *Sarcoptes scabiei* at different stages of development treated with 1 mg/mL RGFP966 and Elevenostat.

RGFP966 and elevenostat (1 mg/mL) were used to target distinct phases of the life cycles of scabies mite. The results indicated a substantial difference between the two inhibitors in terms of killing nymph mites compared with adult mites (*P* < 0.01). However, no significant difference in the killing effect of the two inhibitors was found in the larvae and nymph mites (*P* > 0.05). The differences between the larvae and adult mites were highly significant (*P* < 0.0001) (Fig. 6).

### Transmission electron microscopy observation

**Fig 7:**
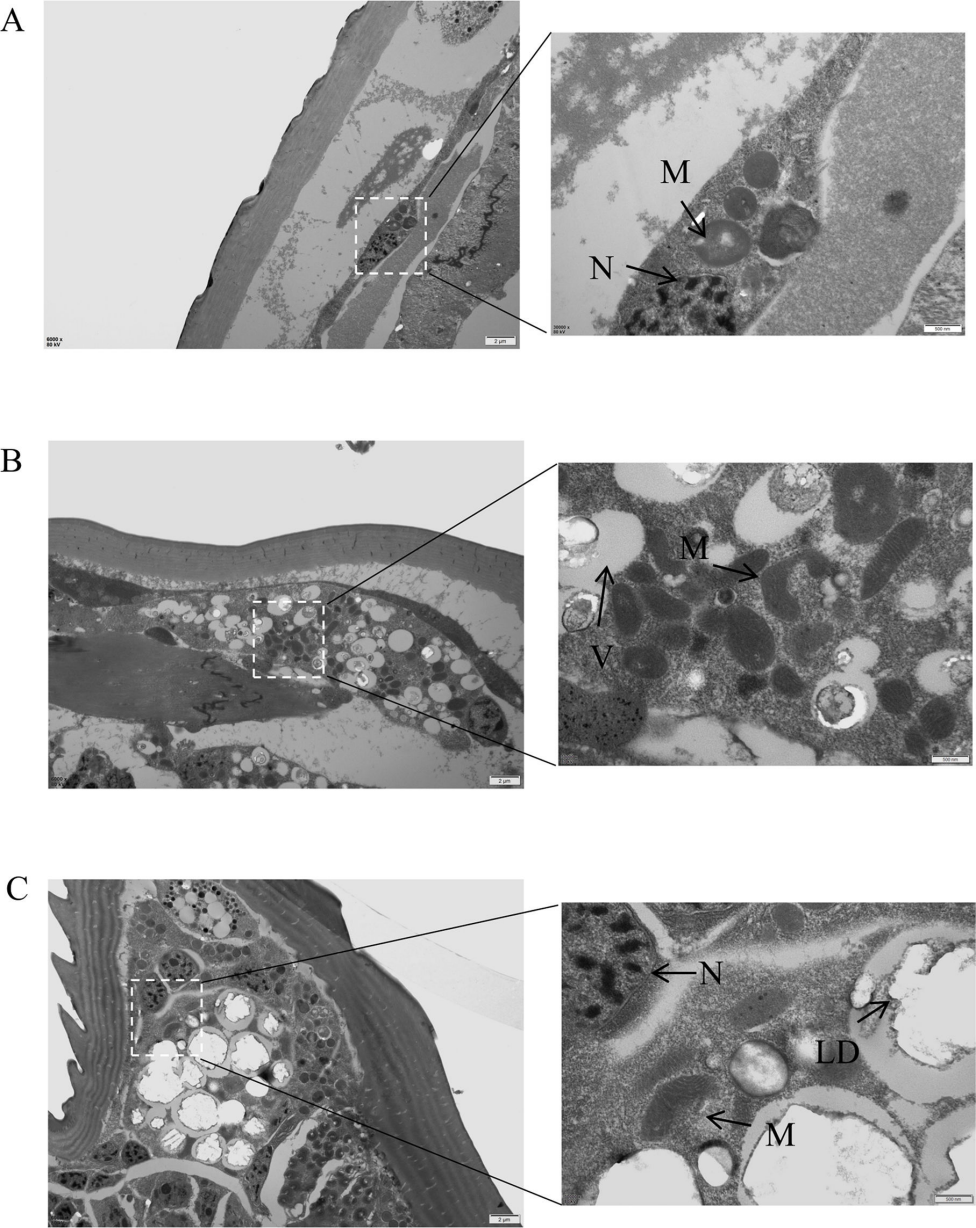
Ultrastructural changes of *Sarcoptes scabies* after 12 h treatment with RGFP966 and Elevenostat. (**A**)10% DMSO; (**B**)1 mg/mL RGFP966; (**C**)1 mg/mL Elevenostat. Notes: N, nucleus; M, mitochondria; V, vacuole; LD, lipid droplet. Scale bars: A, B, and C = 2 µm; A, B, and C insets = 500 nm.

The result TEM showed that the body wall of the mites was smooth, the cytoplasm was tight, and the mitochondrial structure was normal in the negative control group. The individual cells showed mitochondrial pyknosis, cytoplasm dissolution, vacuole formation, and enlarged internuclear spaces after treatment with 1 mg/mL RGFP966. Treatment with 1 mg/mL elevenostat led to stratum corneum shrinkage (Fig. S7), increased intracellular lipid droplets, and mitochondrial pyknosis (Fig. 7).

## DISCUSSION

HDAC-2 is implicated in many tumor disorders, biological barriers, lipid metabolism, genome stability, and cell cycle progression. However, HDAC-3 is more closely linked to energy and lipid metabolism-related activities in life (12). Comprehensive investigation using *Sarcoptes scabiei* β-actin as the reference gene demonstrated that the transcription levels of SsHDAC-2 and SsHDAC-3 differed at each stage based on the quantitative polymerase chain reaction results. The findings indicate that SsHDAC-2 and SsHDAC-3 in *Sarcoptes scabiei* may play a role in the various stages of their growth and development. SsHDAC-2 transcription levels were higher in the nymph stage than in the larvae and adult stages, whereas SsHDAC-3 transcription levels were approximately five times higher in the adult stage than in the larvae and nymph stages. Nymph and female mites had comparable survival rates and typically outlived larvae and male mites (23); SsHDAC-2 was mainly involved in energy acquisition and other life functions in the nymph mites. However, a separate comparison of male and female mites was not performed in this study. More research is required on the gene transcription associated with the female mite stage. SsHDAC-3 was more closely related to lipid and energy metabolism. The transcription level of SsHDAC-3 was significantly higher than that of other stages, possibly because adult mites were proportionally larger and required more energy to survive (24).

Immunofluorescence results showed that SsHDAC-2 was mainly distributed in the anus and intestine of scabies mites. It was speculated that SsHDAC-2 was involved in the regulation of the excretion and secretion process of scabies mites or as an excretion and secretion protein to trigger host immune response. The intestines of scabies mites mainly digested and absorbed nutrients from the host, and the higher distribution of SsHDAC-2 in the intestine might be attributed to its role in transforming nutrients into energy (25). SsHDAC-3 was found in the gastrointestinal tract of scabies mites, as well as in their mouthparts, chelicerae, and body surfaces. This was consistent with earlier research indicating that HDAC-3 was broadly expressed and played an essential role in protein deacetylation (12). HDAC-3 was the key regulator of gene expression and stage transformation in *Toxoplasma gondii* (26). Therefore, it is speculated that H3 may be involved in the growth and development process of scabies mites. Simultaneously, HDAC-3 was implicated in inflammation regulation and played a key role in the inflammatory gene expression program (27). It was hypothesized that SsHDAC-3 was involved in infecting the host and generating inflammatory response.

Elevenostat and RGFP966 were chosen as inhibitors in this study to investigate the role of HDAC in the survival process of scabies mites. The survival activity of the inhibitors against live scabies mites was tested *in vitro*. The acaricidal effects of the two inhibitors were discovered to be time dependent, with maximum fatality rates of 76.7% and 73.3%, respectively, after 24 h of activity at a concentration of 1 mg/mL. The acaricidal effect of elevenostat was also concentration dependent. Although some mites did not die during the early stages of the inhibitor’s action, they did lose their capacity to move, implying that they could no longer infect new hosts. On this basis, we used two inhibitors to incubate scabies mites in various stages of development. The mortality of nymph and larvae after incubation with elevenostat or RGFP966 was much higher than that of adult mites. This was possibly because the nymph and larvae had a higher surface area/volume ratio, allowing them to absorb more inhibitors via the cuticle during incubation (21). Mites could also survive for extended periods of time at lower temperatures and higher relative humidity. Adult mites tended to survive longer than nymph and young mites when the temperature was lower, but when the temperature was higher than 30°C, the survival time of adult mites was the shortest (28). This experiment was conducted at 25°C, which might be one of the reasons for the relatively higher survival rate of adult mites.

TEM was used to examine the effects of RGFP966 and elevenostat on the ultrastructure of mites. It was discovered that the scabies mites suffered changes in mitochondrial pyknosis, cytoplasm disintegration to create vacuoles, and formation of intracellular lipid droplets. Similar results were observed when broad-spectrum HDAC inhibitors were used against *T. gondii* and *Trypanosoma* (26, 29, 30). HDAC-3 regulated mitochondrial activity and metabolism, as well as reactive oxygen species-generating enzymes, antioxidant enzymes, and oxidative stress-related transcription factors (31). HDAC-11 (SsHDAC-2 homologous sequence) was also found in mitochondria and was involved in mitochondrial fatty acid oxidation (32). This suggested that RGFP966 and elevenostat caused mitochondrial changes in scabies mites by inhibiting HDAC-2 and HDAC-3. Pyknosis was a significant feature of apoptosis, and HDAC inhibitors could increase the transcription levels of cysteine proteinase 3 and 7 when treated with Schistosoma, and it has also been demonstrated that suppression of HDAC-3 and HDAC-11 influences the degree of apoptosis (33–35) . Therefore, we hypothesized that the mitochondria of scabies mites induced apoptosis through cysteine proteinase-dependent pathway after treatment with RGFP966 and elevenostat. The development of vacuoles was linked to cell cycle arrest and autophagy. The HDAC inhibitor MC1742 could cause cyclin-dependent kinase overexpression and cell cycle defects in *T. gondii* merozoites, and MHY218 could induce autophagy (36, 37). Both inhibitors showed an increase in the number of vacuoles in the cytoplasm, indicating that SsHDAC-2 and SsHDAC-3 might regulate mite cell cycle and autophagy. HDAC-3 and HDAC-11 played roles in controlling cellular energy metabolism and oxidative stress. The inhibitors produced stress in mite cells and increased the number of lipid droplets, which could be related to mitochondrial condensation. Lipid buildup occurred in *Trypanosoma cruzi* treated with HDAC inhibitors, which could be related to the changes in cell cycle progression (38, 39). Elevenostat therapy results in scabies mite cuticle reduction, and inhibition of parasite HDAC could also alter cytoskeletal proteins (26). It has been documented that cytoskeletal protein changes result from the inhibition of parasite HDAC and that α-tubulin deacetylation is also affected by HDAC-11 suppression (26, 40). Therefore, it is speculated that after elevenostat incubates scabies mites, it may cause the inhibition of SsHDAC-2, thereby affecting the deacetylation of α-tubulin in the scabies mite skeleton protein, resulting in damage to the mite body. According to this study, it can speculate that Elevenostat and RGFP966 can inflict structural damage on mites by inhibiting SsHDAC-2 and SsHDAC-3, thereby compromising their survival capabilities. This discovery offers a novel approach to the treatment of scabies.

## Data Availability

The data sets used and/or analyzed are detailed in the supplemental information files. The supplemental material for this article can be found at the following URL: https://doi.org/10.5061/dryad.15dv41p5m. Any further information is available from the corresponding author on reasonable request.
